# Supplementary material for: Unraveling the electrocatalytic reduction mechanism of enols on copper in aqueous media
Source: Nat Commun. 2022 Oct 3;13:5840. doi: 10.1038/s41467-022-33620-2 (PMC9530228; doi:10.1038/s41467-022-33620-2)
Supplement: Supplementary file 1 — Supplementary Information [file 41467_2022_33620_MOESM1_ESM.pdf]

Supporting Information

to

Unraveling the Electrocatalytic Reduction Mechanism of Enols on Copper in Aqueous Media

Zhihao Cui[1], Xing'an Dong[2], Sung Gu Cho[1], Modeste N. Tegomoh[1], Weidong Dai[2], Fan Dong[2], Anne C. Co [1*]*

1 Department of Chemistry and Biochemistry, The Ohio State University, Columbus, Ohio 43210, United States

2 Research Center for Environmental & Energy Catalysis, Institute of Fundamental and Frontier Sciences, University of Electronic Science and Technology of China, Chengdu 611731, China.

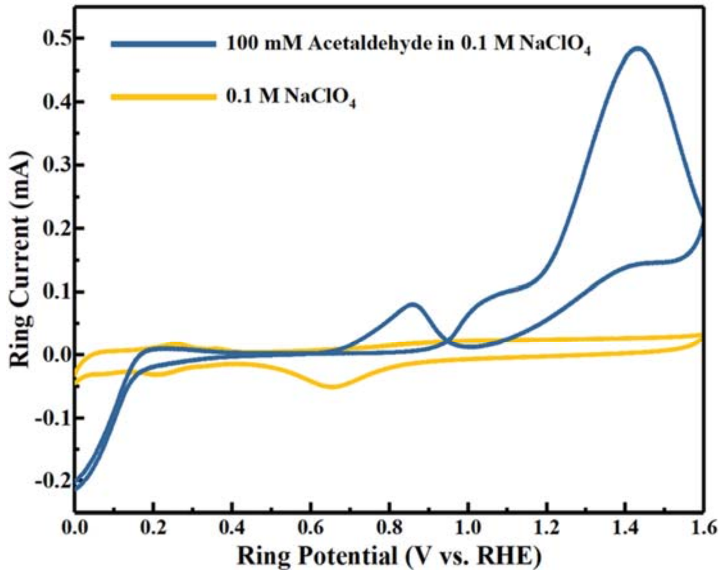

Supplementary Figure 1. CV at Pt ring in the presence of acetaldehyde. Electrolyte: 0.1 M $NaClO_4$ containing 100 mM acetaldehyde. CV conditions: 0 to 1.6 V and then back to 0 V (vs RHE), 100 mV/s, 1600 RPM, 298 K.

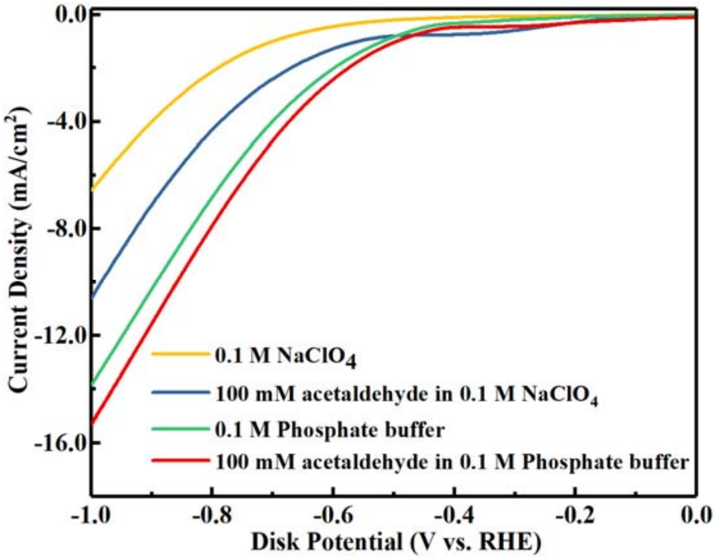

Supplementary Figure 2. Linear sweep voltammetry (LSV) for acetaldehyde reduction on Cu. Electrolyte: 0.1 M $NaClO_4$ and sodium phosphate buffer (pH = 7) containing 100 mM acetaldehyde. LSV conditions: 0 to -1.0 V and then back to 0 V (vs RHE), 10 mV/s, 1600 RPM, 298 K. The current densities are normalized by electrode geometric areas.

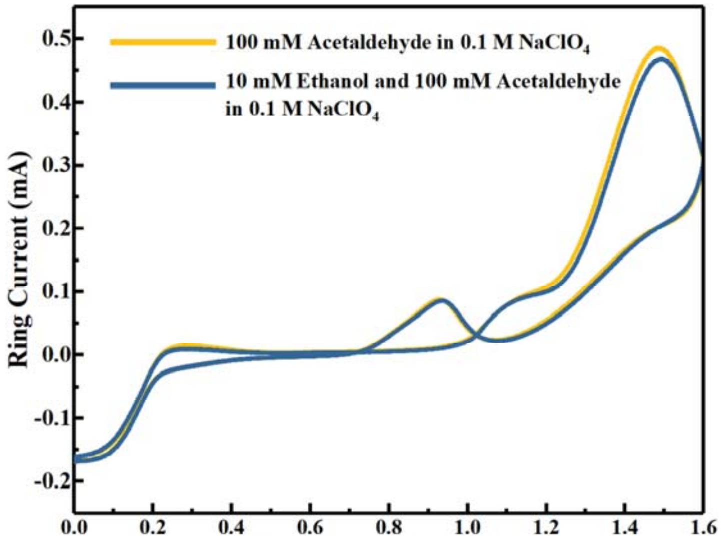

Supplementary Figure 3. CV at Pt ring in the presence of acetaldehyde and ethanol. Electrolyte: 0.1 M $NaClO_4$ containing 10mM Ethanol and 100 mM acetaldehyde. CV conditions: 0 to 1.6 V and then back to 0 V (vs RHE), 100 mV/s, 1600 RPM, 298 K.

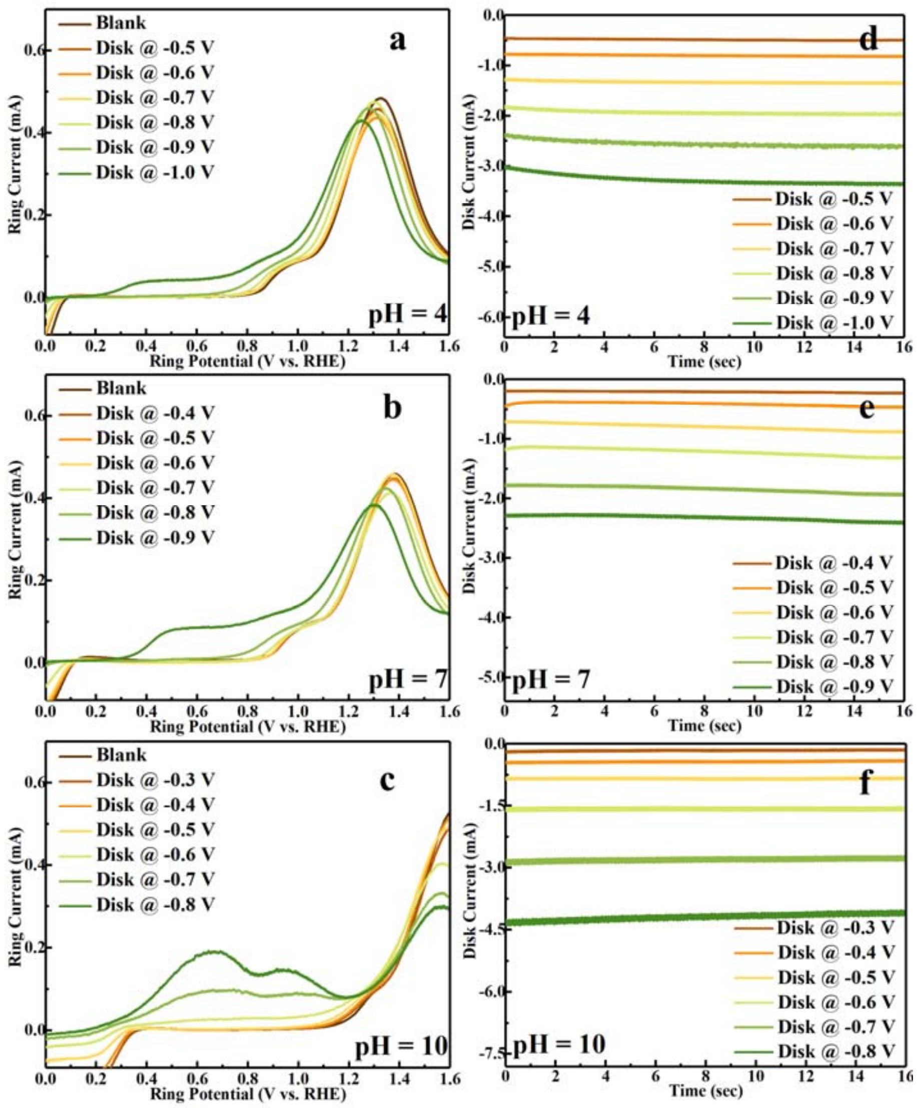

Supplementary Figure 4. Control experiment in 0.1 M phosphate buffer solution. a,b,c Cyclic voltammograms (CVs) of products detected by the Pt ring electrode during Cu catalyzed acetaldehyde reduction at pH = 4, 7, 10, respectively. Only the positive-going scans were shown for clarity. CV of the acetaldehyde blank was collected on the Pt ring when Cu disk was at open circuit potential (OCP). **d,e,f** Total Cu disk current during acetaldehyde reduction. CV conditions: 0 to 1.6 V and then back to 0 V (vs. RHE), 100 mV/s, 1600 RPM, 298 K.

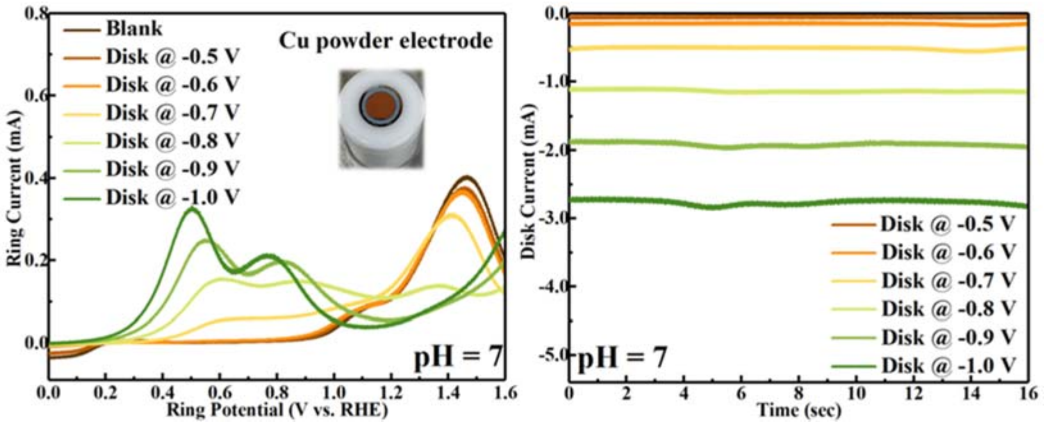

Supplementary Figure 5. Control experiment by using Cu powder as working electrode. Left: Cyclic voltammograms (CVs) of products detected by the Pt ring electrode during Cu powder catalyzed acetaldehyde (100 mM) reduction at pH = 7 (0.1 M $NaClO_4$). Only the positive-going scans were shown for clarity. Cu powder electrode potential was held from -0.5 V to -1.0 V. CV of the acetaldehyde blank was collected on the Pt ring when Cu powder electrode was at open circuit potential. Right: Total Cu powder electrode current during acetaldehyde reduction. CV conditions: 0 to 1.6 V and then back to 0 V (vs. RHE), 100 mV/s, 1600 RPM, 298 K.

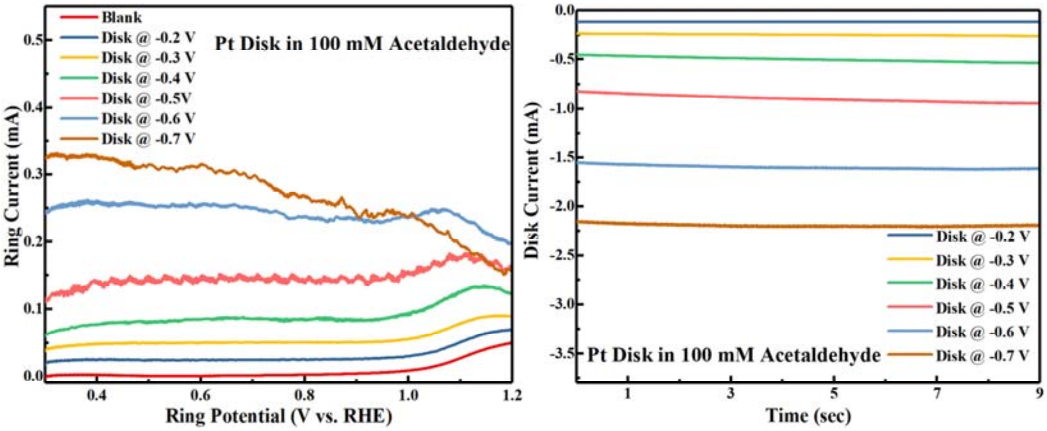

Supplementary Figure 6. RRDE measurements during acetaldehyde reduction on Pt disk electrode. Electrolyte: 0.1 M $NaClO_4$ containing 100 mM acetaldehyde. CV conditions: 0 to 1.6 V and then back to 0 V (vs RHE), 100 mV/s, 1600 RPM, 298 K. Only the positive-going scans were shown in CVs for clarity.

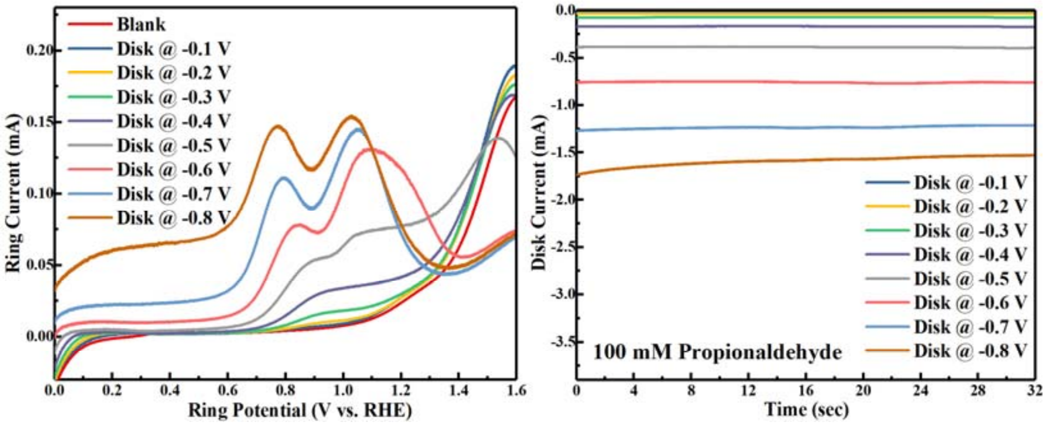

Supplementary Figure 7. RRDE measurements during propionaldehyde reduction on Cu disk electrode. Electrolyte: 0.1 M $NaClO_4$ containing 100 mM propionaldehyde. CV conditions: 0 to 1.6 V and then back to 0 V (vs RHE), 50 mV/s, 1600 RPM, 298 K. Only the positive-going scans were shown in CVs for clarity.

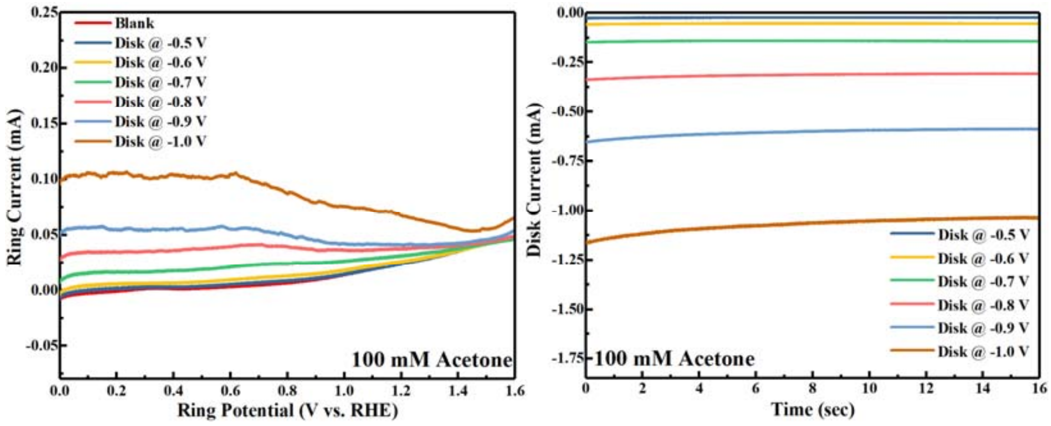

Supplementary Figure 8. RRDE measurements during acetone reduction on Cu disk electrode. Electrolyte: 0.1 M $NaClO_4$ containing 100 mM acetone. CV conditions: 0 to 1.6 V and then back to 0 V (vs RHE), 100 mV/s, 1600 RPM, 298 K. Only the positive-going scans were shown in CVs for clarity.

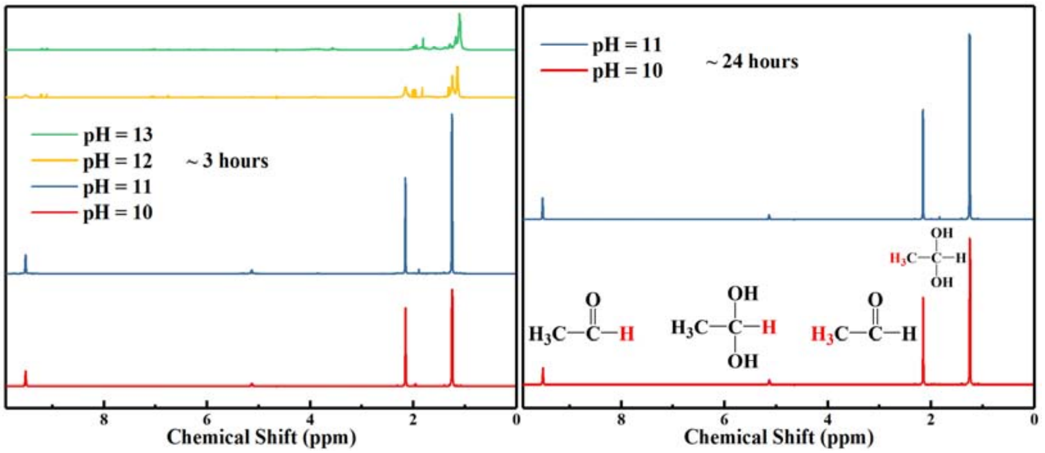

Supplementary Figure 9. ^{1}H NMR spectra of 100 mM acetaldehyde in $NaClO_4$ solutions under different pH conditions. These spectra were collected after aging acetaldehyde solution at indicated conditions for 3 hours, 24 hours, respectively.

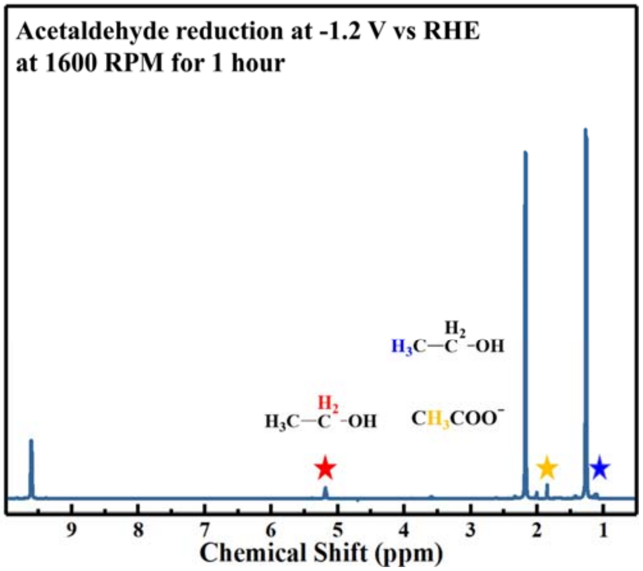

Supplementary Figure 10. ^{1}H NMR spectra of liquid products from reduction of 100 mM acetaldehyde on RDE-Cu disk in 0.1 M $NaClO_4$ electrolyte solution. The spectrum was collected right after RDE-GC-MS measurement at indicated condition.

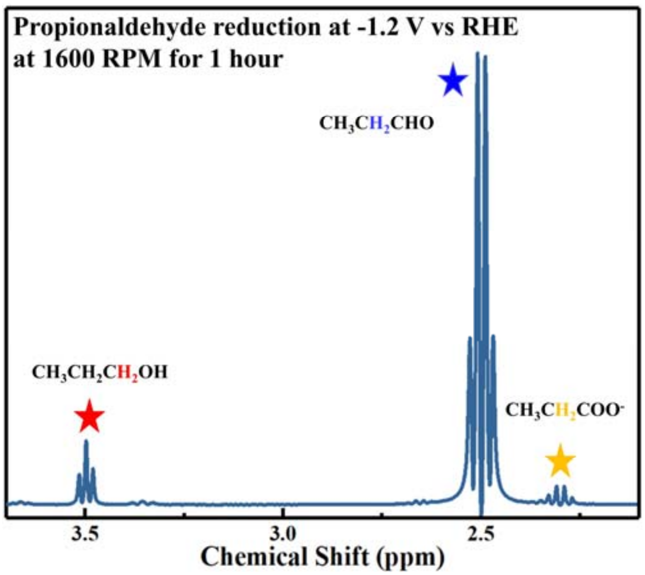

Supplementary Figure 11. ^{1}H NMR spectra of liquid products from reduction of 100 mM propionaldehyde on RDE-Cu disk in 0.1 M $NaClO_4$ electrolyte solution. The spectrum was collected right after RDE-GC-MS measurement at indicated condition.

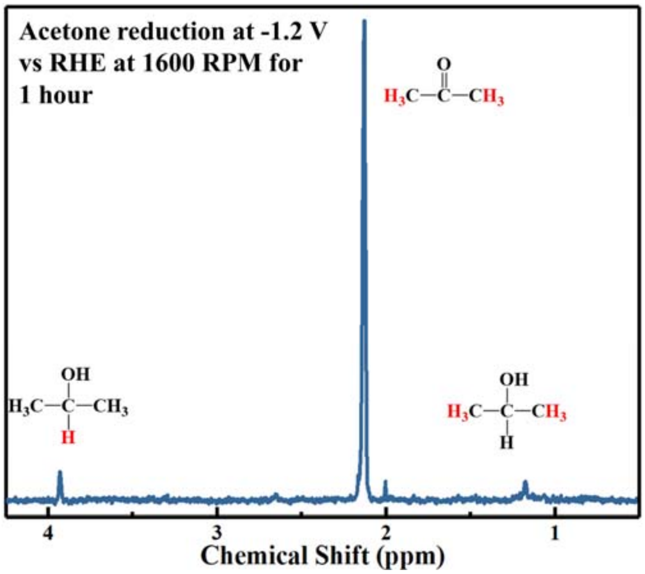

Supplementary Figure 12. ^{1}H NMR spectra of liquid products from reduction of 100mM acetone on RDE-Cu disk in 0.1 M $NaClO_4$ electrolyte solution. The spectrum was collected right after RDE-GC-MS measurement at indicated condition.

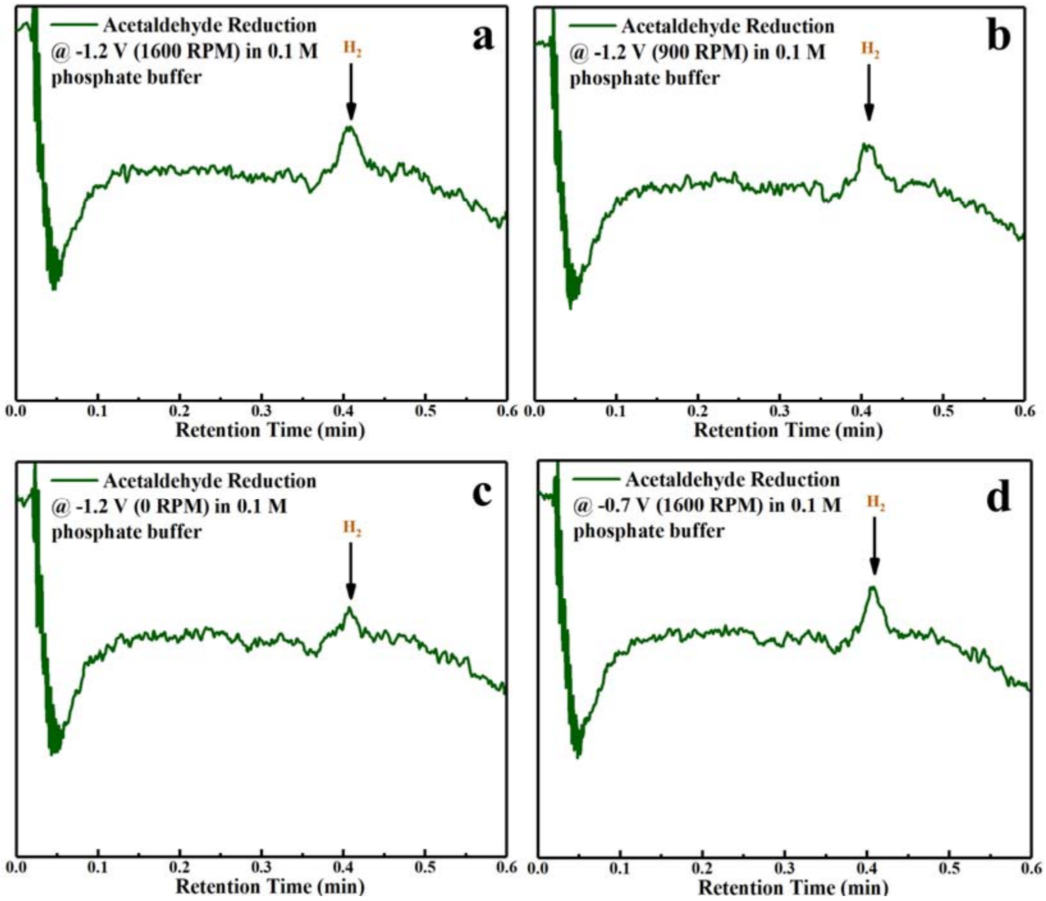

Supplementary Figure 13. Control experiment for hydrogen detection in buffer solution. Gaseous products generated from the electroreduction of acetaldehyde. Gas products were auto-injected via a gas sampling mechanism and analyzed using GC-MS in selected ion monitoring (SIM) mode at: a -1.2 V vs. RHE, 1600 RPM; b -1.2 V vs. RHE, 900 RPM; c -1.2 V vs. RHE, no rotation; d -0.7 V vs. RHE, 1600 RPM in 0.1 M phosphate buffer solution saturated with N_2(pH=7).

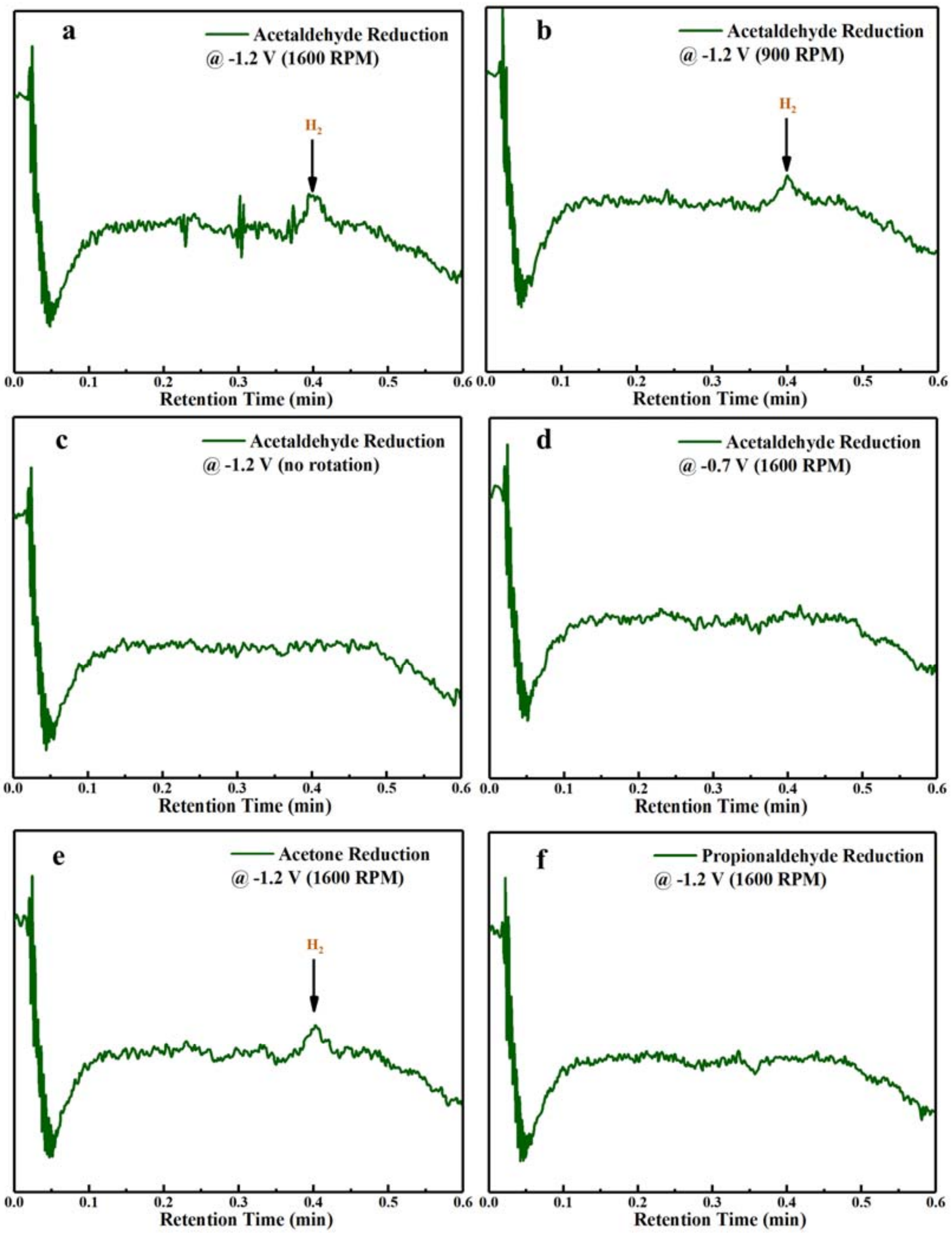

Supplementary Figure 14. RDE-GC-MS measurements from thermal conductivity detector (TCD). Acetaldehyde reduction at -1.2 V vs RHE, (a) 1600 RPM; (b) 900 RPM; (c) no rotation; (d) Acetaldehyde reduction at -0.7 V vs RHE, 1600 RPM; (e) Acetone reduction at -1.2 V vs RHE, 1600 RPM; (f) Propionaldehyde reduction at -1.2 V vs RHE, 1600 RPM, in 0.1 M $NaClO_4$ solution saturated with N_2(pH=7).

76

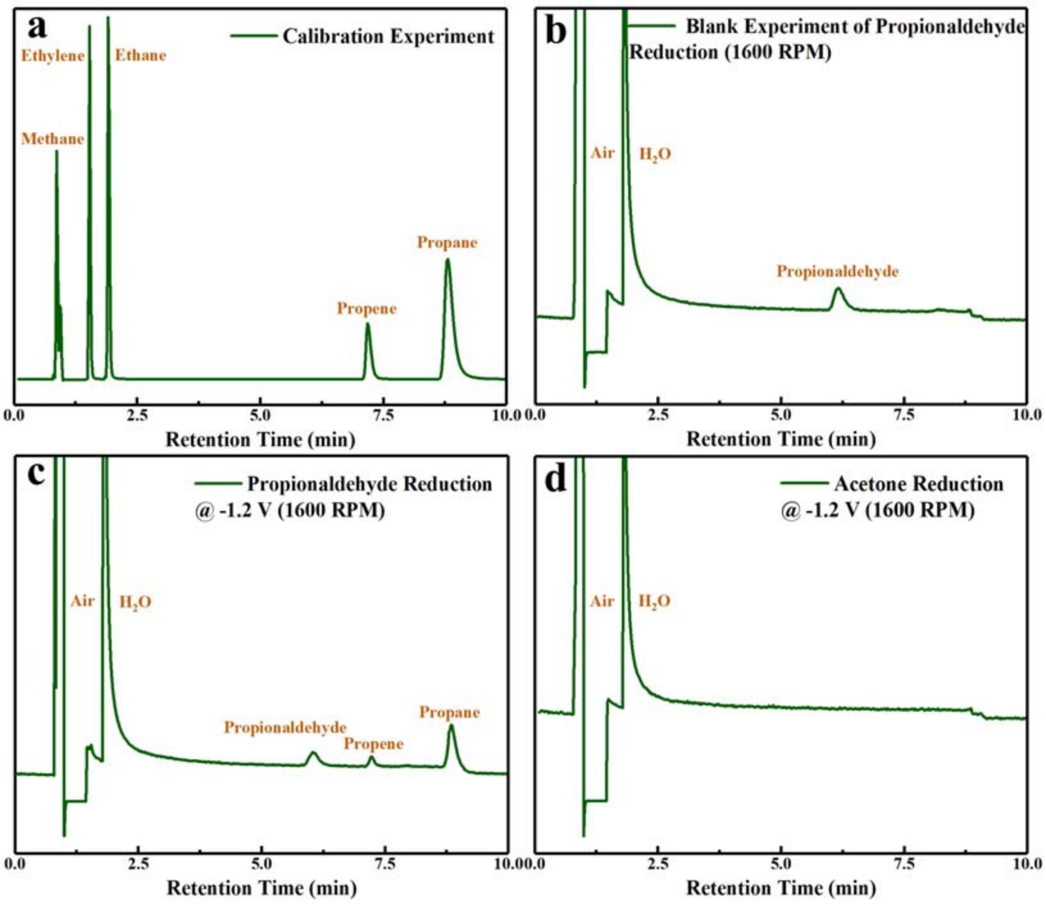

Supplementary Figure 15. RDE-GC-MS measurements. (a) Calibration experiment; (b) Blank experiment for propionaldehyde reduction; (c) Gaseous products detection during reduction of propionaldehyde; (d) Gaseous products detection during reduction of acetone, in 0.1 M $NaClO_4$ solution saturated with N_2(pH=7).

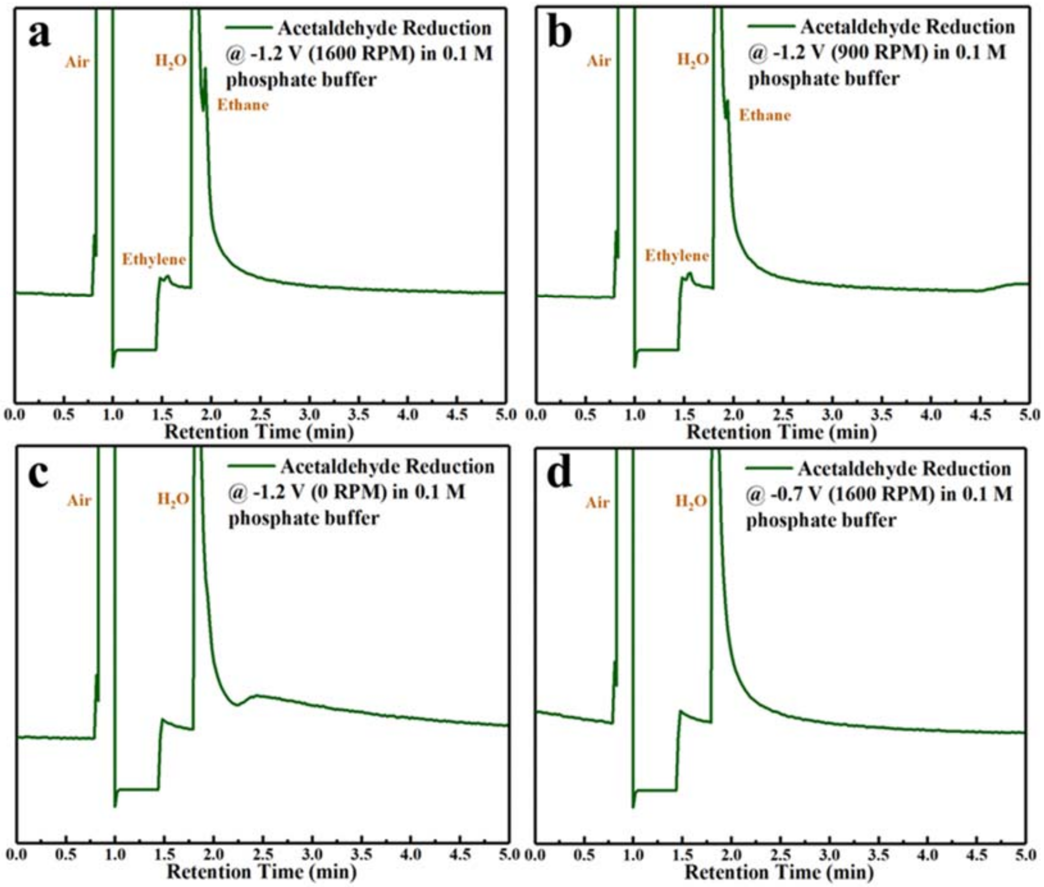

Supplementary Figure 16. Control experiment for hydrocarbons detection in buffer solution. Gaseous products generated from the electroreduction of acetaldehyde. Gas products were auto-injected via a gas sampling mechanism and analyzed using GC-MS in selected ion monitoring (SIM) mode at: a -1.2 V vs. RHE, 1600 RPM; b -1.2 V vs. RHE, 900 RPM; c -1.2 V vs. RHE, no rotation; d -0.7 V vs. RHE, 1600 RPM in 0.1 M phosphate buffer solution saturated with N_2(pH=7).

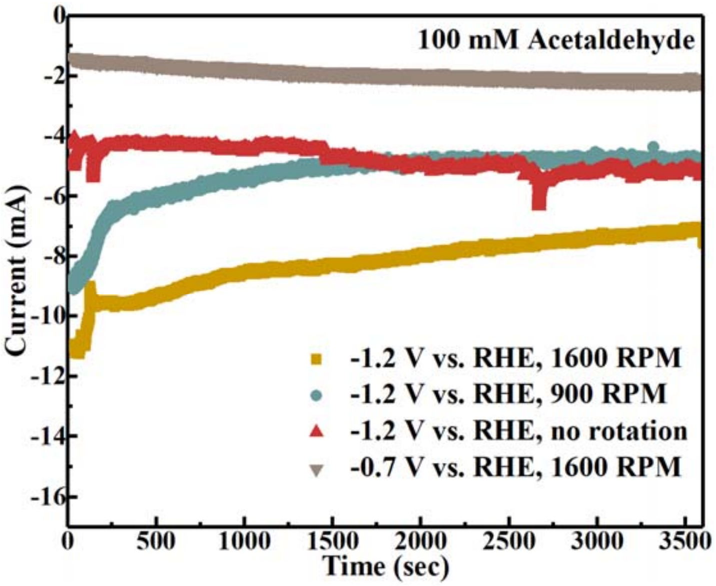

Supplementary Figure 17. Total current for the acetaldehyde reduction over Cu disk in 0.1 M $NaClO_4$ at indicated conditions.

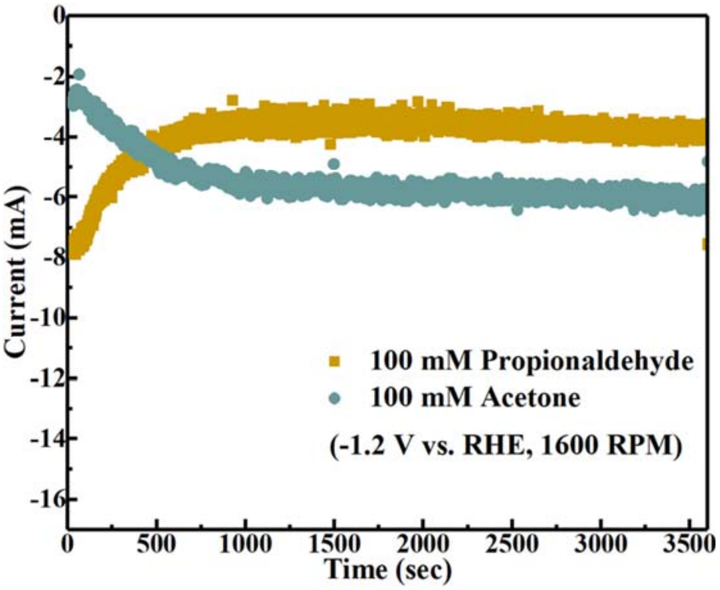

Supplementary Figure 18. Total current for the propionaldehyde and acetone reduction over Cu disk in 0.1 M $NaClO_4$ at indicated conditions.

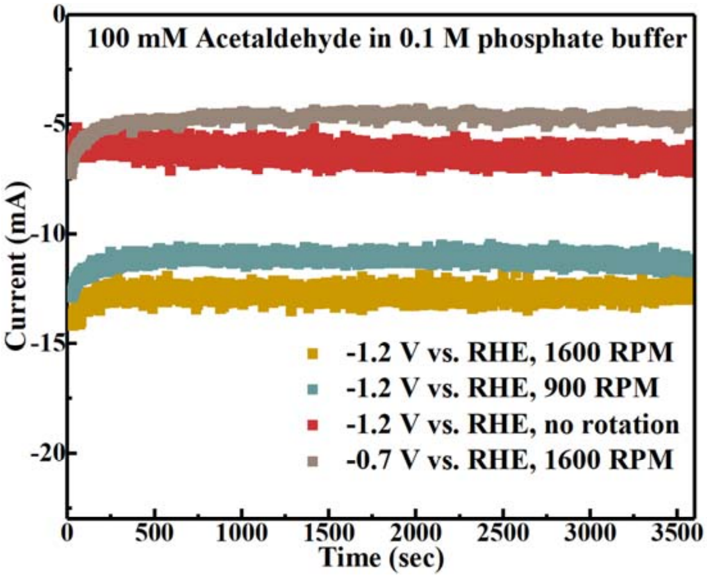

Supplementary Figure 19. Control experiment for total current for the acetaldehyde reduction over Cu disk in buffer solution. 0.1 M phosphate buffer solution saturated with N_2(pH=7).

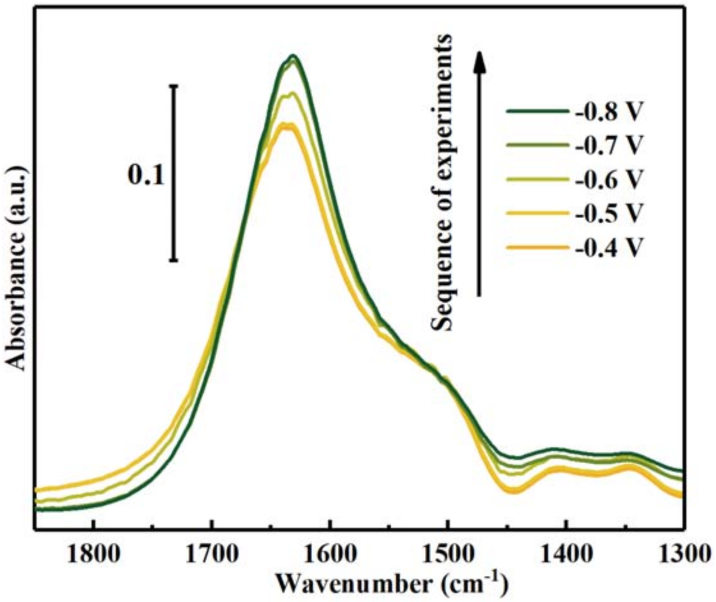

Supplementary Figure 20. Additional in situ ATR-SEIRAS spectra obtained on polycrystalline copper as a function of potentials in Argon saturated 0.1 M $NaClO_4$ (pH=7) with 100 mM acetaldehyde. All the potentials are on the RHE scale.

Supplementary Note 1. H/D exchange experiment

To make our band assignments more reliable, in situ Raman tests were conducted in D_2O based electrolyte solution as shown in Supplementary Fig.21. Two bands appeared at 2183 cm^{-1} and 2111 cm^{-1} at -0.3 V in D_2O which are assigned to the symmetrical C-D stretching of CD_3 and CD_2 groups of the adsorbed ethoxy intermediate [1,2]. These assignments can be further supported by Raman bands located at lower frequency region. As highlighted in yellow region, two bands at 885 cm^{-1} and 847 cm^{-1} at -0.3 V in D_2O can be assigned to the rocking vibration of CD_3 group [4]. The band at 636 cm^{-1} at -0.3 V in D_2O is assigned to the rocking vibration of CD_2 group [5-7]. These groups may have been formed via a surface H/D exchange process as reported in a previous study [8]. Collectively, the shifts in the Raman bands supports the assignment of 2800-3000 cm^{-1} bands to adsorbed ethoxy, as opposed to the presence of an exogenous species.

More importantly, OH group of vinyl alcohol will be completely deuterated if we assume a fast H/D exchange between the OH group of vinyl alcohol and D_2O. Considering that tautomerization of acetaldehyde to vinyl alcohol is slow then it is possible that even adsorbed vinyl alcohol can be reduced to ethoxy on Cu surface at -0.3 V. Bands between 2100-2200 cm^{-1} are assigned to CD_2 and CD_3 groups at -0.3 V in D_2O. This result is consistent with previous computational studies on Cu(100) [9,10] which show that electroreduction of adsorbed vinyl alcohol to ethoxy is thermodynamically downhill at -0.3 V vs RHE. However, more well-designed experiments should be conducted to further support this argument and this will be the focus of our future work.

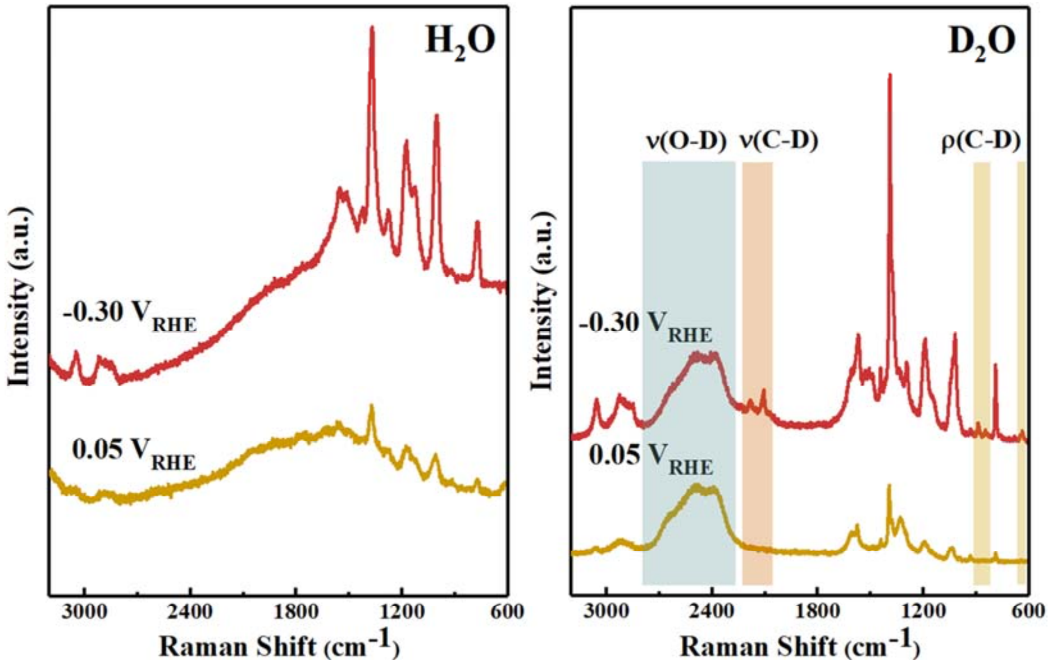

Supplementary Figure 21. Additional in situ Raman spectra obtained on polycrystalline copper powder electrode as a function of potential in Ar saturated 0.1 M $NaClO_4$ (pH=7) with 100 mM acetaldehyde. The electrolyte is based on H_2O (left) and D_2O (right).

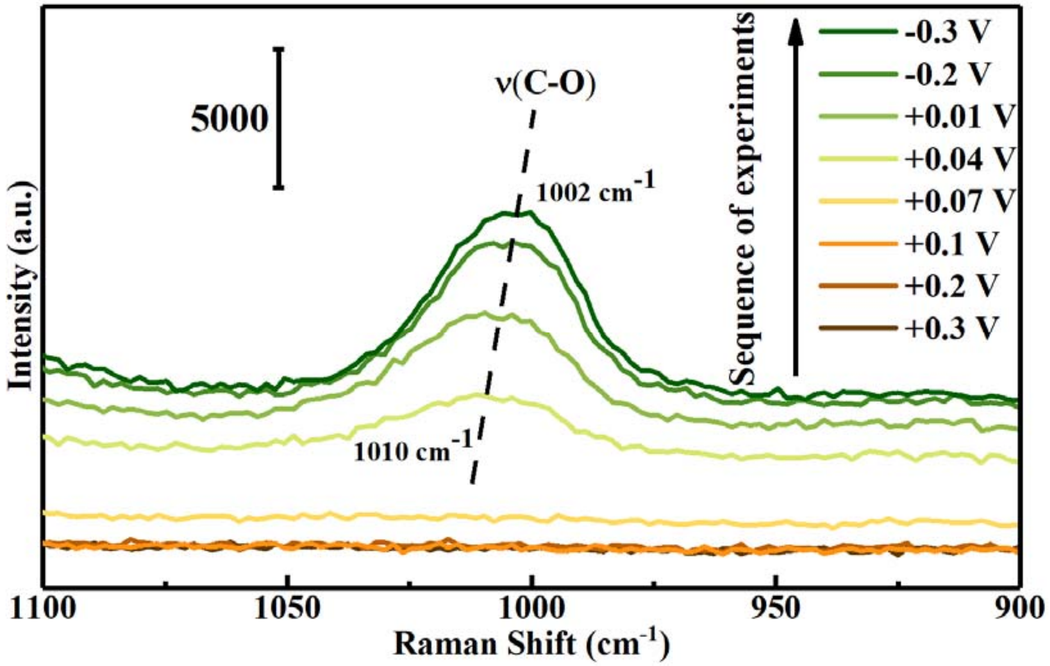

Supplementary Figure 22. In situ Raman spectra of the C-O stretching vibration region obtained on polycrystalline copper as a function of potential in Ar saturated 0.1 M $NaClO_4$ (pH=7) with 100 mM acetaldehyde. All the potentials are on the RHE scale.

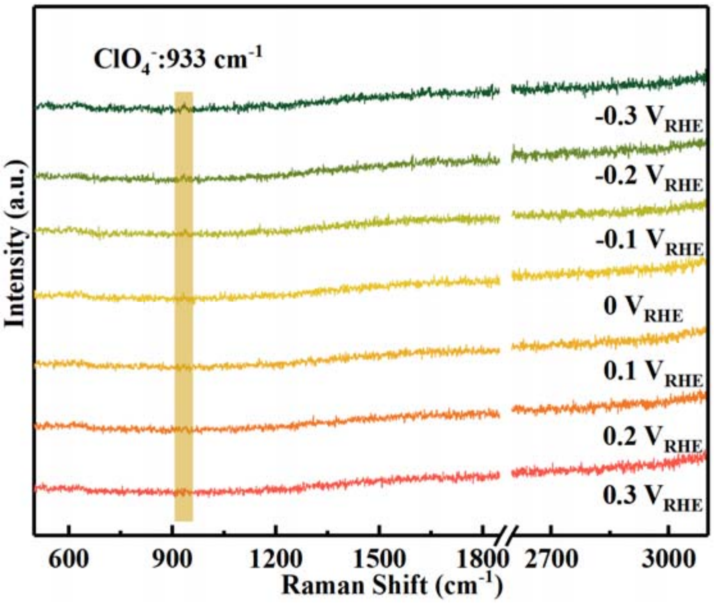

Supplementary Figure 23. Blank in situ Raman spectra obtained on polycrystalline copper as a function of potentials in Ar saturated 0.1 M $NaClO_4$ (pH=7).

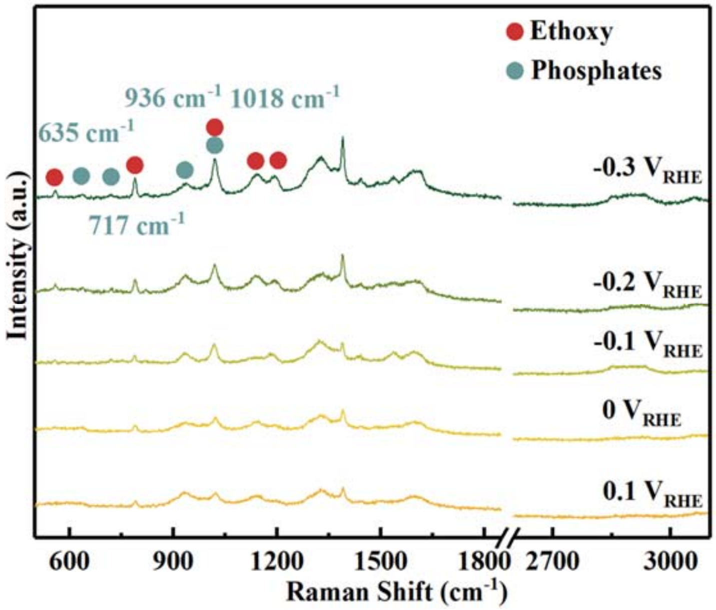

Supplementary Figure 24. Control experiment for in-situ Raman experiment in 0.1 M phosphate buffer solution (pH = 7) with an applied potential ranging between -.1 to -0.3 V vs. RHE.

Supplementary Note 2. In situ Raman experiment in 0.1 M phosphate buffer solution

According to a previous surface enhanced Raman spectroscopy (SERS) study on phosphate anions adsorption on Cu electrode [11], each band between 500 and 1200 cm^{-1} was labeled to differentiate the bands resulted from adsorbed phosphates and ethoxy. The band around 635 cm^{-1} can be assigned to asymmetrical stretching vibration of PO_4^{3-}. The small band at 717 cm^{-1} is assigned to stretching vibration from $H_2PO_4^-$ species. It is important to note that primary phosphate adsorption band is located at 936 cm^{-1}, which can be assigned to P-O* (O* denotes adsorbed oxygen) stretching vibration from PO_4^{3-}, HPO_4^{2-} or $H_2PO_4^-$. It is possible that P-O stretching vibration band from $H_2PO_4^-$ overlaps with C-O stretching band of ethoxy at 1018 cm^{-1}, so we assign the band at 1018 cm^{-1} to both ethoxy and phosphate. It's important to note that local pH changes may result in the variation of intensities of phosphate bands because in situ Raman detects not only adsorbed species but also local species near the electrode surface [12].

In the phosphate buffer solution, vibrational band at 3046 cm^{-1}, 2920 cm^{-1} and 2860 cm^{-1} appeared at ca. 0 V_{RHE}, which is also observed at the potential values of 0.04 V_{RHE} and 0.07 V_{RHE} obtained in 0.1 M $NaClO_4$ solution. These bands were assigned to adsorbed vinyl alcohol and ethoxy intermediate, respectively. As a result, we can conclude that the same adsorbed intermediates are present in the phosphate buffer solution at a similar electrode potential on Cu.

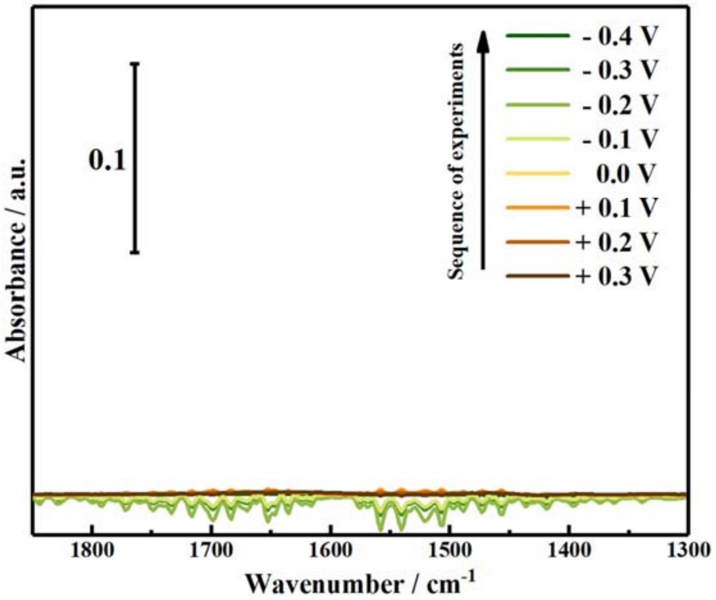

Supplementary Figure 25. In situ ATR-SEIRAS spectra of pure Au film deposited on Si. Spectra were recorded at the same experimental condition to exclude the influence from Au film in probing the intermediates during acetaldehyde reduction.

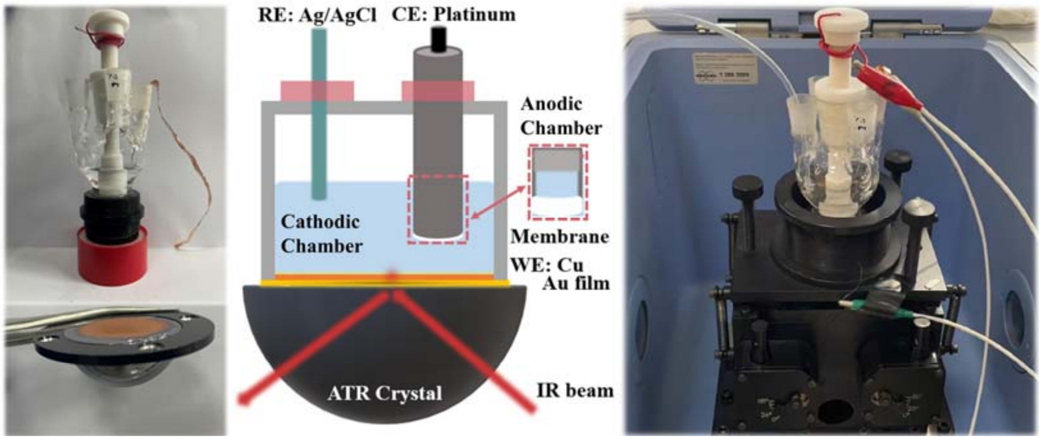

Supplementary Figure 26. A schematic of ATR-SEIRAS setup consisting of two chambers with three electrodes.

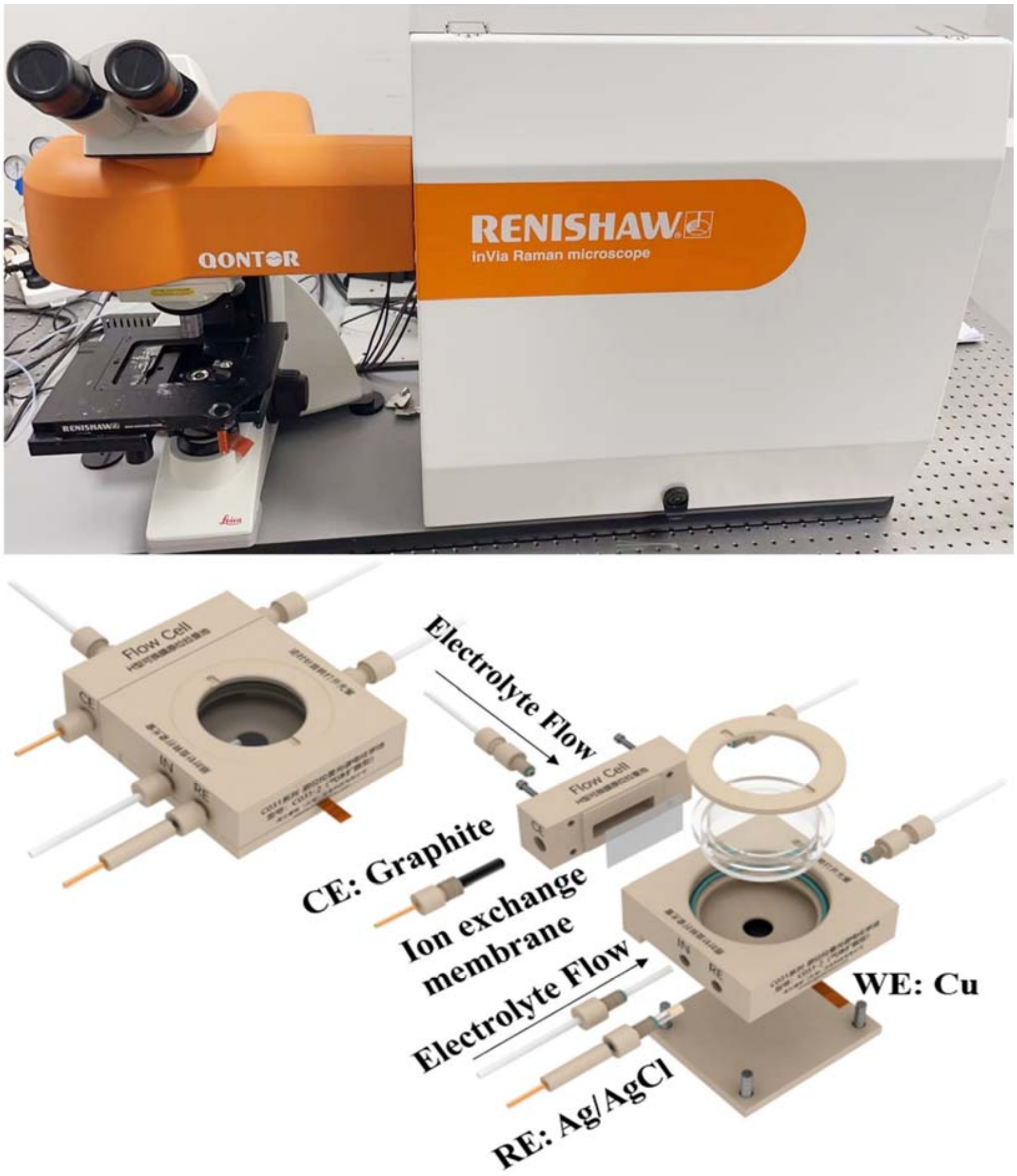

Supplementary Figure 27. A photograph of in situ Raman setup (Renishaw-RL633) and a schematic of flow cell used in in situ Raman test. A similar cell was also used in previous Raman based study reported by other group [3].

Supplementary Note 3. Additional Computational Information

All thermodynamic corrections for various gaseous species used in this study is provided in Supplementary Table 1. The heat capacities and entropies of $CH_3CHO(g)$ and $CH_3CH_2OH(g)$ are taken from standard thermodynamic tables [13,14].

Supplementary Table 1. Zero-point energies, heat capacities, and entropies for gas phase molecules are included at p = 1 atm and T = 298.15 K. All values are given in eV.

$Species$	ZPE	$\int C_p dT$	TS
$H_2(g)$	0.27	0.09	0.40
$H_2O(g)$	0.57	0.10	0.58
$C_2H_4(g)$	1.35	0.11	0.68
$C_2H_6(g)$	1.98	0.16	0.71
$CH_3CHO(g)$	1.46	0.17	0.82
$CH_3CH_2OH(g)$	2.11	0.20	0.87

To estimate the free energies for substances in liquid-phase, we use previously reported thermodynamic workaround [15-17]. Generally, the free energy of liquid substance can be calculated by adding the difference between the free energies of formation of its liquid and gas phases to the gas phase entropy. Such differences are 0.09 eV, 0.07 eV, -0.06 eV for water, ethanol and acetaldehyde, respectively. The free energy of liquid vinyl alcohol is estimated according to experimental reported pK_e value of 6.23 [18] for the tautomerization equilibrium between acetaldehyde and vinyl alcohol in aqueous solution at 298 K, so we have the following expression:

$$\Delta G^{l}_{Keto/Enol} = G^{l}_{Vinyl\ alcohol} - G^{l}_{Acetaldehyde} = 0.37\ eV$$

Supplementary Table 2. Zero-point energies, heat capacities, entropies and solvation corrections for adsorbed species on Cu(100) are included at T = 298.15 K. All values are given in eV.

$Species$	ZPE	$\int C_p dT$	TS	$E_{solvation}$	Ref
$*CH_2{=}CHOH$	1.51	0.17	0.09	-0.38	[17]
$*CH{=}CH_2{+}*OH$	1.43	0.14	0.26	-0.58	[17]
$*CH{=}CH_2$	1.08	0.09	0.16	N/A	N/A
$*CH{=}CH_3$	1.36	0.10	0.20	N/A	N/A
$*CH_2{=}CH_3$	1.67	0.11	0.22	N/A	N/A
$*CH_3CHOH$	1.81	0.14	0.28	-0.38	[17]
$*CH_2CH_2OH$	1.84	0.13	0.25	-0.38	[17]

Supplementary Table 3. Zero-point energies, heat capacities, entropies and solvation corrections for adsorbed species on Cu(211) are included at T = 298.15 K. All values are given in eV.

$Species$	ZPE	$\int C_p dT$	TS	$E_{solvation}$	Ref
$*CH_2{=}CHOH$	1.51	0.09	0.16	-0.38	[17]
$*CH{=}CH_2{+}*OH$	1.43	0.14	0.25	-0.58	[17]
$*CH{=}CH_2$	1.09	0.08	0.17	N/A	N/A
$*CH{=}CH_3$	1.36	0.08	0.14	N/A	N/A
$*CH_2{=}CH_3$	1.67	0.11	0.23	N/A	N/A
$*CH_3CHOH$	1.83	0.13	0.26	-0.38	[17]
$*CH_2CH_2OH$	1.84	0.13	0.27	-0.38	[17]

Supplementary Table 4. Grand-canonical DFT calculated potential dependent free energies of slabs (G_*) and vinyl alcohol adsorbed on the slabs ((G_{M^*}) on Cu(100) and Cu(211). All values are given in Ry.

$Electrode\ Poetntial$ ($V\ vs. SHE$)	$G_*^{Cu(100)}$	$G_{M^*}^{Cu(100)}$	$G_*^{Cu(211)}$	$G_{M^*}^{Cu(211)}$
-1.1	-4372.810	-4432.342	-4372.940	-4432.493
-0.7	-4372.809	-4432.339	-4372.939	-4432.491
-0.3	-4372.808	-4432.338	-4372.938	-4432.489
0.1	-4372.808	-4432.336	-4372.938	-4432.487
0.5	-4372.808	-4432.336	-4372.938	-4432.486

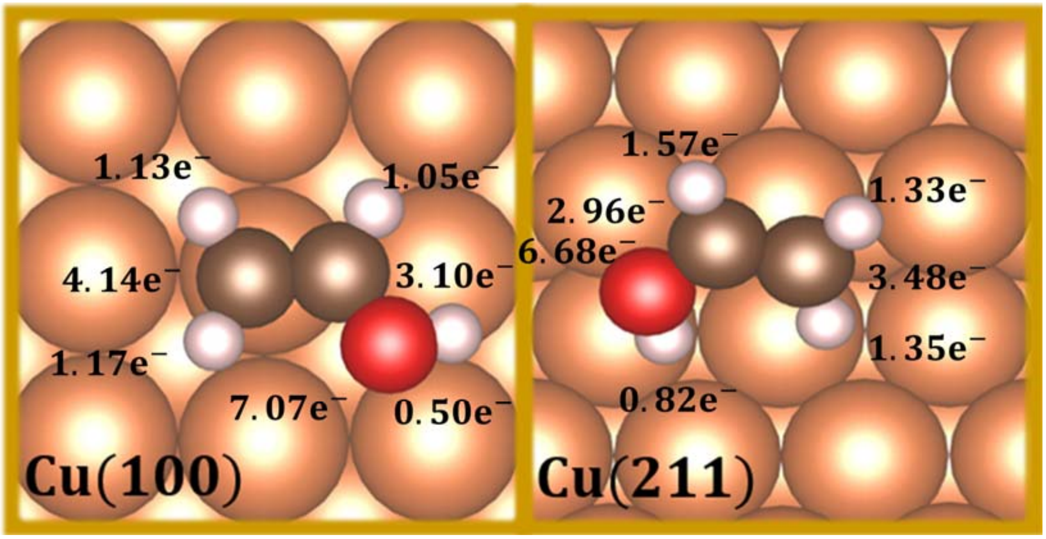

Supplementary Figure 28. Counting of valence electrons per atom for adsorbed vinyl alcohol on Cu(100) and Cu(211), obtained through Bader charge analysis.

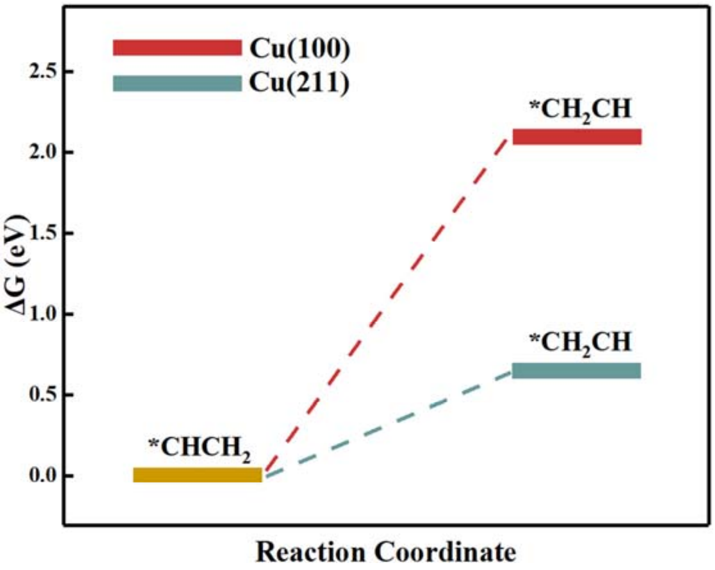

Supplementary Figure 29. Calculated free energy of adsorbed *$CHCH_2$ and *CH_2CH on Cu(100) and Cu(211).

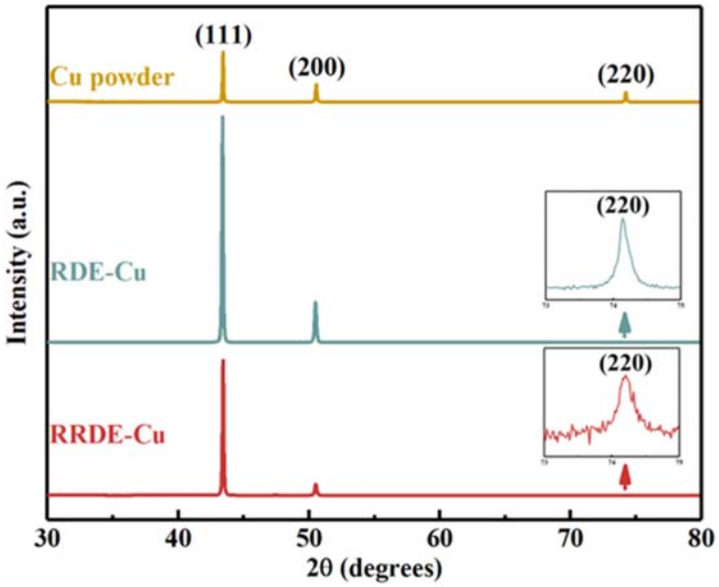

Supplementary Figure 30. X-ray diffraction (XRD) patterns of polycrystalline Cu powder (yellow), RDE-Cu disk (green) and RRDE-Cu disk (red). XRD data was collected on a Bruker D8 Advance A25 diffractometer using a Cu K_α X-ray tube.

Supplementary References

1. Nolin, B. & Jones, R. N. THE INFRARED ABSORPTION SPECTRA OF DEUTERATED ESTERS: II. ETHYL ACETATE. *Canadian Journal of Chemistry* **34**, 1392-1404, doi:10.1139/v56-178 (1956).
2. Westre, S. G. & Kelly, P. B. Examination of CD_3 vibrational structure by resonance Raman spectroscopy. *The Journal of Chemical Physics* **90**, 6977-6979, doi:10.1063/1.456273 (1989).
3. Zhao, Y. *et al.* Speciation of Cu Surfaces During the Electrochemical CO Reduction Reaction. *Journal of the American Chemical Society* **142**, 9735-9743, doi:10.1021/jacs.0c02354 (2020).
4. Lang, S. M., Bernhardt, T. M., Bakker, J. M., Yoon, B. & Landman, U. Methanol C–O Bond Activation by Free Gold Clusters Probed via Infrared Photodissociation Spectroscopy. **233**, 865-880, doi:doi:10.1515/zpch-2018-1368 (2019).
5. Sawodny, W., Niedenzu, K. & Dawson, J. W. The vibrational spectrum of ethylene glycol. *Spectrochimica Acta Part A: Molecular Spectroscopy* **23**, 799-806, doi:https://doi.org/10.1016/0584-8539(67)80007-2 (1967).
6. Sakakini, B., Harendt, C. & Vickerman, J. C. An EELS study of the adsorption and decomposition of deuterated ethene on Cu/Ru(0001). *Spectrochimica Acta Part A: Molecular Spectroscopy* **43**, 1613-1618, doi:https://doi.org/10.1016/S0584-8539(87)80057-0 (1987).
7. Mendelsohn, R., Davies, M. A., Schuster, H. F., Xu, Z. & Bittman, R. CD_2 rocking modes as quantitative infrared probes of one-, two-, and three-bond conformational disorder in dipalmitoylphosphatidylcholine and dipalmitoylphosphatidylcholine/cholesterol mixtures. *Biochemistry* **30**, 8558-8563, doi:10.1021/bi00099a010 (1991).
8. Bondue, C. J. & Koper, M. T. M. A mechanistic investigation on the electrocatalytic reduction of aliphatic ketones at platinum. *Journal of Catalysis* **369**, 302-311, doi:https://doi.org/10.1016/j.jcat.2018.11.019 (2019).
9. Luo, W., Nie, X., Janik, M. J. & Asthagiri, A. Facet Dependence of CO_2 Reduction Paths on Cu Electrodes. *ACS Catalysis* **6**, 219-229, doi:10.1021/acscatal.5b01967 (2016).
10. Santatiwongchai, J., Faungnawakij, K. & Hirunsit, P. Comprehensive Mechanism of CO_2 Electroreduction toward Ethylene and Ethanol: The Solvent Effect from Explicit Water–Cu(100) Interface Models. *ACS Catalysis* **11**, 9688-9701, doi:10.1021/acscatal.1c01486 (2021).
11. Niaura, G., Gaigalas, A. K. & Vilker, V. L. Surface-Enhanced Raman Spectroscopy of Phosphate Anions: Adsorption on Silver, Gold, and Copper Electrodes. *The Journal of Physical Chemistry B* **101**, 9250-9262, doi:10.1021/jp970097k (1997).
12. Yang, K., Kas, R. & Smith, W. A. In Situ Infrared Spectroscopy Reveals Persistent Alkalinity near Electrode Surfaces during CO_2 Electroreduction. *Journal of the American Chemical Society* **141**, 15891-15900, doi:10.1021/jacs.9b07000 (2019).
13. CRC Handbook of Chemistry and Physics, 87th ed Editor-in-Chief: David R. Lide (National Institute of Standards and Technology). CRC

Press/Taylor and Francis Group: Boca Raton, FL. 2006. 2608 pp. $139.95. ISBN 0-8493-0487-3. *Journal of the American Chemical Society* **129**, 724-724, doi:10.1021/ja069813z (2007).

14 Chase, M. W., Jr. *NIST-JANAF thermochemical tables, monograph 9*. Fourth Edition edn, Vol. 9 (American Chemical Society, 1998).

15 Ledezma-Yanez, I., Gallent, E. P., Koper, M. T. M. & Calle-Vallejo, F. Structure-sensitive electroreduction of acetaldehyde to ethanol on copper and its mechanistic implications for CO and CO2 reduction. *Catalysis Today* **262**, 90-94, doi:https://doi.org/10.1016/j.cattod.2015.09.029 (2016).

16 Peterson, A. A., Abild-Pedersen, F., Studt, F., Rossmeisl, J. & Nørskov, J. K. How copper catalyzes the electroreduction of carbon dioxide into hydrocarbon fuels. *Energy & Environmental Science* **3**, 1311-1315, doi:10.1039/C0EE00071J (2010).

17 Calle-Vallejo, F. & Koper, M. T. M. Theoretical Considerations on the Electroreduction of CO to C2 Species on Cu(100) Electrodes. *Angewandte Chemie International Edition* **52**, 7282-7285, doi:https://doi.org/10.1002/anie.201301470 (2013).

18 Keeffe, J. R., Kresge, A. J. & Schepp, N. P. Keto-enol equilibrium constants of simple monofunctional aldehydes and ketones in aqueous solution. *Journal of the American Chemical Society* **112**, 4862-4868, doi:10.1021/ja00168a035 (1990).
